# The Role of Inulin in Maintaining Antioxidant Capacity and Enzymatic Activities of Jerusalem Artichoke (*Helianthus tuberosus* L.) Cultivars During Cold Storage

**DOI:** 10.3390/antiox14091109

**Published:** 2025-09-12

**Authors:** Yuwen Mu, Bohua Zhang, Shiqi Lv, Fencan Li, Changming Zhao

**Affiliations:** 1 Agricultural Product Storage and Processing Research Institute, Gansu Academy of Agricultural Sciences, Lanzhou 730070, China; 2 State Key Laboratory of Herbage Improvement and Grassland Agro-Ecosystems, College of Ecology, Lanzhou University, Lanzhou 730000, China; 3 Yuzhong Mountain Ecosystems Observation and Research Station, Lanzhou University, Lanzhou 730000, China; 4 Jinan Fruit Research Institute, All China Federation of Supply & Marketing Co-Operatives, Jinan 250014, China

**Keywords:** Jerusalem artichoke, inulin, polyphenol, antioxidant capacity, enzyme activities, cold storage

## Abstract

Jerusalem artichoke (*Helianthus tuberosus* L.) is valued for its high inulin content and adaptability to marginal lands. This study investigated the changes in inulin content, antioxidant capacity, polyphenol concentrations, and enzymatic activities of eight cultivars during 60 days of cold storage. Inulin levels ranged from 582.43 g/kg (LZJ006) to 809.70 g/kg (LZJ055), with LZJ047 maintaining the highest content throughout storage. The antioxidant potential, as measured by ferric reducing antioxidant power (FRAP) and 2,2-diphenyl-1-picrylhydrazyl (DPPH) assay, declined across all cultivars, correlating with the reduction in inulin content. The polyphenol content varied significantly, with LZJ119 having 2.17 times more than LZJ010. POD activity increased, while catalase (CAT) and superoxide dismutase (SOD) activities fluctuated during the storage period. Hierarchical Cluster Analysis identified three distinct antioxidant clusters, revealing significant correlations between inulin content and key antioxidant parameters (CAT, FRAP, DPPH). These findings highlight the pivotal role of inulin in preserving the antioxidant system and bioactive properties of Jerusalem artichoke tubers during extended cold storage, providing valuable insights for post-harvest management and cultivar selection.

## 1. Introduction

Jerusalem artichoke (*Helianthus tuberosus* L.), a member of the Asteraceae family and the genus *Helianthus* (sunflower), is a perennial crop native to the north-central United States [1]. It is highly adaptable, thriving in marginal lands such as poor, sandy soils and saline–alkali environments. Due to its resilience, Jerusalem artichoke produces substantial quantities of carbohydrates, making it a valuable crop [2]. Its tubers, along with the above-ground parts of the plant, have diverse industrial applications, including use in food, animal feed, chemicals, and bioenergy production [3,4]. The tubers are particularly notable for their high levels of inulin and polyphenols, compounds known for their health-promoting properties such as prebiotic, antioxidant, and anti-inflammatory effects [5]. Consequently, Jerusalem artichoke is recognized as a valuable source of dietary fiber and natural antioxidants, making it an important functional food ingredient [6].

Previous research indicates that the concentrations of inulin and polyphenols in Jerusalem artichoke tubers can vary significantly depending on factors such as cultivar, agronomic practices, harvest timing, and postharvest storage conditions [7,8,9,10]. Despite its nutritional benefits, Jerusalem artichoke tubers are susceptible to substantial postharvest losses, primarily due to softening, sprouting, inulin depolymerization, and pathogen-induced decay. As a result, preserving key compounds like inulin and polyphenols during storage is essential for maintaining tuber quality and extending their shelf life.

Among the various postharvest techniques, low-temperature storage is recognized as one of the most effective methods for preserving the quality of Jerusalem artichoke tubers, helping to reduce metabolic activity and delay deterioration [11]. Storage at temperatures between 0 and 2 °C with high relative humidity can extend the tubers’ storage life up to 6–12 months. However, the influence of such conditions on the preservation of bioactive compounds, particularly inulin and polyphenols, remains less well understood. These compounds not only contribute to the tubers’ nutritional value but are also crucial for their functional properties, including antioxidant activity, which plays a key role in preventing oxidative damage.

With growing consumer demand for functional foods with health benefits, it is increasingly important to retain bioactive compounds in foods during storage. The storage conditions, particularly temperature and humidity, play a critical role in determining the stability of inulin, polyphenols, and other key nutrients in Jerusalem artichoke tubers. Optimizing these conditions is essential for maintaining the tubers’ nutritional and functional qualities during storage.

The health benefits of fruit and vegetables are well-established, largely due to the antioxidants they contain, which help prevent chronic diseases [12,13]. Phytochemicals like polyphenols are central to this defense, protecting plant cells from oxidative stress by neutralizing reactive oxygen species (ROS). These interactions mitigate oxidative damage and help stabilize cellular structures [14,15,16]. Thus, polyphenols are essential for maintaining cellular homeostasis under stress conditions.

In addition to phytochemicals, antioxidant enzymes are critical to the plant defense system, acting as ROS scavengers that protect plants from environmental challenges such as pathogen attacks, mechanical injuries, extreme temperatures, and radiation [17]. Key antioxidant enzymes include catalase (CAT), superoxide dismutase (SOD), and ascorbate peroxidase (APX), all of which play essential roles in managing oxidative stress. For instance, CAT catalyzes the decomposition of hydrogen peroxide, preventing its accumulation, while SOD and APX help detoxify superoxide radicals and hydrogen peroxide, respectively [18,19]. The coordinated action of these enzymes represents a crucial mechanism for plants to withstand oxidative stress.

While previous studies have investigated the impact of postharvest storage on inulin content in Jerusalem artichoke, there is a lack of comprehensive research examining the changes in inulin alongside the broader antioxidant systems across different cultivars during storage. Furthermore, the role of inulin’s degree of polymerization (DP) in modulating the antioxidant capacity and enzymatic activities remains largely unexplored. This study aims to fill these knowledge gaps by providing new insights into the dynamic relationships between inulin content, its DP, polyphenol concentrations, and antioxidant enzyme activities in eight Jerusalem artichoke cultivars from a semi-arid region of China during cold storage.

The innovative aspects of this research lie in its holistic approach to understanding the complex interactions between inulin, polyphenols, and antioxidant enzymes in Jerusalem artichoke tubers under low-temperature storage conditions. By employing high-performance liquid chromatography (HPLC) to determine the DP of inulin and correlating it with antioxidant capacity, this study provides a novel perspective on the functional role of inulin beyond its well-characterized molecular structure. Additionally, the comparative analysis of eight cultivars from a semi-arid region of China contributes to the identification of superior genotypes with enhanced bioactive properties and storage stability, which can inform future breeding programs and postharvest management strategies.

We hypothesize that changes in both inulin content and its DP during cold storage will significantly affect the antioxidant capacity and related enzymatic activities in Jerusalem artichoke tubers. By elucidating these relationships, this study aims to advance our understanding of the mechanisms underlying the biochemical resilience of Jerusalem artichoke tubers and optimize postharvest storage conditions to preserve their bioactive properties. The findings of this research have the potential to guide the development of targeted strategies for maintaining the nutritional and functional quality of Jerusalem artichoke tubers during storage, ultimately benefiting both producers and consumers in the rapidly growing functional food market.

## 2. Materials and Methods

### 2.1. Materials

All chemical reagents were of analytical grade or higher purity unless otherwise specified.

Reagents for inulin analysis: Sodium hydroxide (≥98%, CAS 1310-73-2) and sodium acetate (≥99%, CAS 127-09-3) were purchased from Shanghai Aladdin Bio-Chem Technology Co., Ltd. (Shanghai, China) for mobile phase preparation in HPAEC-PAD analysis.

Reagents for total polyphenol determination: Folin–Ciocalteu reagent, anhydrous sodium carbonate (≥99.5%, CAS 497-19-8), and gallic acid (≥98%, CAS 149-91-7) as standard were obtained from Shanghai Yuanye Bio-Technology Co., Ltd. (Shanghai, China). HPLC-grade methanol (≥99.9%, CAS 67-56-1) was purchased from Shanghai Aladdin Bio-Chem Technology Co., Ltd. (Shanghai, China).

Reagents for antioxidant capacity assays: 2,2-Diphenyl-1-picrylhydrazyl (DPPH, ≥95%, CAS 1898-66-4), 2,4,6-tripyridyl-s-triazine (TPTZ, ≥98%, CAS 3682-35-7), ferric chloride hexahydrate (FeCl_3_·6H_2_O, ≥97%, CAS 10025-77-1), and Trolox (6-hydroxy-2,5,7,8-tetramethylchroman-2-carboxylic acid, ≥97%, CAS 53188-07-1) were purchased from Shanghai Yuanye Bio-Technology Co., Ltd. Disodium EDTA (≥99%, CAS 6381-92-6), hydrogen peroxide (30% w/w, CAS 7722-84-1), L-ascorbic acid (≥99%, CAS 50-81-7), 2-deoxy-D-ribose (≥98%, CAS 533-67-5), thiobarbituric acid (≥98%, CAS 504-17-6), trichloroacetic acid (≥99%, CAS 76-03-9), hydrochloric acid (ACS grade, 37%, CAS 7647-01-0), and glacial acetic acid (CAS 64-19-7) were obtained from Sinopharm Chemical Reagent Co., Ltd. (Shanghai, China).

Reagents for antioxidant enzyme activity assays: Triton X-100 (laboratory grade, CAS 9002-93-1), polyvinylpolypyrrolidone (PVPP, cross-linked type, CAS 25249-54-1), polyvinylpyrrolidone (PVP, average molecular weight 40,000, CAS 9003-39-8), dithiothreitol (DTT, ≥98%, CAS 3483-12-3), guaiacol (≥98%, CAS 90-05-1), L-methionine (≥99%, CAS 63-68-3), nitro blue tetrazolium (NBT, ≥98%, CAS 298-83-9), and riboflavin (≥97.5%, CAS 83-88-5) were purchased from Sinopharm Chemical Reagent Co., Ltd. (Shanghai, China).

Buffer preparation reagents: Potassium dihydrogen phosphate (KH_2_PO_4_, ≥99%, CAS 7778-77-0) and dipotassium hydrogen phosphate (K_2_HPO_4_, ≥99%, CAS 7758-11-4) were obtained from Sinopharm Chemical Reagent Co., Ltd. for phosphate buffer preparation.

All solutions were prepared using deionized water with a resistivity of ≥18.2 MΩ·cm.

### 2.2. Sample Preparation and Treatments

The Jerusalem artichoke (*Helianthus tuberosus* L.) tubers used in this study were obtained from eight cultivars (LZJ004, LZJ005, LZJ006, LZJ010, LZJ017, LZJ047, LZJ055, and LZJ119) grown at the germplasm resource nursery of Lanzhou University (35°56′ N, 104°09′ E, 1750 m asl), Gansu Province, China. These cultivars are maintained at the Germplasm Resource Nursery of Lanzhou University (registration numbers LZJ001–LZJ200) and are available upon reasonable request to the corresponding author. All cultivars were planted in mid-April 2021 under identical field conditions, with a row spacing of 0.8 m and a plant spacing of 0.5 m, using a completely randomized block design with three replications.

The experimental site soil was classified as loessial soil with a pH of 7.3, organic matter content of 1.5%, available nitrogen of 68 mg/kg, available phosphorus of 15 mg/kg, and available potassium of 120 mg/kg. The experimental area is located in the semi-arid region of Northwest China with a continental monsoon climate, average annual temperature of 6.5 °C, and annual precipitation of 320 mm, of which over 60% occurs between July and September.

The tubers were harvested simultaneously in late November 2021, following the senescence of the aboveground plant parts, to ensure a valid comparison among cultivars. Prior to harvest, the experimental field experienced several mild frost events with minimum temperatures between −2 and −4 °C, lasting for approximately two weeks. Tuber sorting was based on size uniformity (length 5–8 cm), absence of mechanical damage, disease symptoms, or sprouting. Only intact, healthy tubers were selected for experiments to ensure consistency between samples.

Selected tubers were pre-cooled at 2 °C and stored in polyethylene bags at 0 ± 0.5 °C with 90–95% relative humidity. Each cultivar was divided into three replicates, with each replicate consisting of 2 kg of tubers. Samples were systematically collected at intervals of 0, 30, and 60 days. After collection, tubers were thoroughly washed with tap water, surface dried, and then diced into approximately 0.5 × 0.5 × 0.5 cm cubes using a stainless-steel dicer. The diced samples were rapidly flash-frozen in liquid nitrogen to preserve their biochemical integrity and stored at −80 °C until further analysis. The moisture content of fresh tubers ranged from 78.2% to 82.5% across all cultivars, with an average of 80.3 ± 1.8%.

### 2.3. Inulin Analysis

Given that inulin’s structure has been well established in previous studies [1], quantifying inulin content and its degree of polymerization (DP, number average) through high-performance liquid chromatography (HPLC) is an appropriate analytical approach. Inulin was extracted by homogenizing 1 g of the sample in 9 mL of hot distilled water (85 °C) for 1 h, followed by centrifugation at 10,000× *g* for 10 min at 4 °C. The supernatant was analyzed using high-performance anion-exchange chromatography with pulsed amperometric detection (HPAEC-PAD) on a Dionex ICS-5000 ion chromatograph with a CarboPac PA200 column. The inulin content and DP profile were determined following Timmermans et al. with modifications [20]. The analysis used 100 mM NaOH as mobile phase A and 1 M sodium acetate in 100 mM NaOH as mobile phase B, with a flow rate of 0.25 mL/min. The gradient elution program was: 0–5 min, 0% B; 5–40 min, 0–50% B; 40–45 min, 50–0% B; 45–60 min, 0% B. Column temperature was maintained at 30 °C with an injection volume of 25 μL. Detection was performed in PAD mode using the following quadruple waveform: E1 = +0.1 V (t1 = 0–0.4 s, integration 0.2–0.4 s), E2 = -2.0 V (t2 = 0.41–0.42 s), E3 = +0.6 V (t3 = 0.43 s), E4 = −0.1 V (t4 = 0.44–0.5 s).

### 2.4. Determination of Total Polyphenols

Total polyphenols were quantified using the Folin–Ciocalteu method [21]. 2 g of frozen samples were ground into fine powder in liquid nitrogen using a mortar and pestle, then extracted with 20 mL of 60% (*v*/*v*) methanol by shaking at 150 rpm for 1 h at room temperature (25 ± 2 °C), centrifuged at 10,000× *g* for 20 min at 4 °C, and the supernatant was collected. The extract (1 mL) was mixed with 1 mL of 10-fold diluted Folin–Ciocalteu reagent and reacted for 5 min. Then, 3 mL of 7.5% (*w*/*v*) sodium carbonate solution was added, and the mixture was incubated at 40 °C for 30 min. Absorbance was measured at 765 nm using a Varian Cary^®^ 100 UV–vis spectrophotometer (Varian Australia Pty. Ltd., Victoria, Australia). The polyphenol content was expressed in terms of gallic acid equivalents per gram of fresh weight (g GAE kg^−1^).

### 2.5. Measurement of Antioxidant Capacities

#### 2.5.1. 2,2-Diphenyl-1-picrylhydrazyl (DPPH) Assay

The DPPH assay involved mixing 2 mL of the sample extract (prepared as described in Section 2.3 and Section 2.4, respectively) with 2 mL of 20 mmol L^−1^ DPPH (2,2-diphenyl-1-picrylhydrazyl) solution in methanol. After 30 min incubation at room temperature (25 ± 2 °C) in the dark, absorbance was measured at 517 nm using a Varian Cary^®^ 100 UV–vis spectrophotometer (Varian Australia Pty. Ltd., Victoria, Australia) [22]. The DPPH radical scavenging capacity of the sample was calculated using the following Formula (1):

(1) $$\mathrm{DPPH}\;\mathrm{radical}\;\mathrm{scavenging}\;\mathrm{activity}\left(\%\right)=\left[1-\frac{A_{sample}-A_{blank}}{A_{control}}\right]\times 100\%$$

where $A_{sample}$ refers to the absorbance of DPPH solution with sample extract, $A_{blank}$ represents the absorbance of sample extract in methanol without DPPH (for sample color correction), and $A_{control}$ corresponds to the absorbance of DPPH solution with methanol instead of sample extract. All measurements were corrected by subtracting the absorbance of pure methanol in the cuvette. DPPH (Inulin) represents the radical scavenging activity of the isolated inulin fraction, while DPPH (TPC) indicates the scavenging capacity associated specifically with the polyphenol fraction.

#### 2.5.2. Ferric Reducing Antioxidant Power (FRAP) Assay

FRAP activity was evaluated based on the method by Benzie and Strain (1996), with some modifications [23]. The reaction involved mixing 0.1 mL of the extract (prepared as described in Section 2.3 and Section 2.4, respectively) with 2.4 mL of FRAP reagent (The FRAP reagent was freshly prepared by mixing 0.3 M acetate buffer (pH 3.6), 10 mM TPTZ (2,4,6-tripyridyl-s-triazine) in 40 mM HCl, and 20 mM FeCl_3_·6H_2_O solution in a ratio of 10:1:1 (*v*/*v*/*v*)), incubating at 37 °C for 10 min, and measuring absorbance at 593 nm by a Varian Cary^®^ 100 UV–vis spectrophotometer (Varian Australia Pty. Ltd., Victoria, Australia). The antioxidant capacity was determined using a Trolox standard curve, with the results expressed as milligrams of Trolox equivalents per gram of fresh weight (g TE kg^−1^). To determine the specific contribution of different components to total antioxidant capacity, FRAP (Inulin) represents the ferric reducing power of the isolated inulin fraction, while FRAP (TPC) indicates the antioxidant capacity associated with the polyphenol fraction.

#### 2.5.3. ^•^OH Scavenging Assay

The ^•^OH scavenging activity was measured using a modified deoxyribose assay, which evaluates the sample’s ability to compete with deoxyribose for hydroxyl radicals, thereby inhibiting the formation of malondialdehyde from deoxyribose oxidation [24]. The reaction mixture (3 mL) contained 0.2 mL sample extract (prepared as described in Section 2.3 and Section 2.4, respectively), 0.2 mL 10 mM FeCl_3_, 0.2 mL 1 mM EDTA (used to chelate Fe^3+^ and control the Fe^2+^/Fe^3+^ ratio), 0.2 mL 10 mM H_2_O_2_, 0.2 mL 10 mM ascorbic acid, 0.2 mL 10 mM 2-deoxyribose, and 2 mL 50 mM phosphate buffer (pH 7.4). EDTA plays a key role in this assay by controlling iron species, as it chelates Fe^3+^, allowing formation of Fe^2+^-EDTA complex, which reacts with H_2_O_2_ to generate site-specific hydroxyl radicals. After thoroughly mixing, the mixture was incubated in a water bath at 40 °C for 60 min, then 1 mL of 50 g/L trichloroacetic acid and 1 mL of 2 g/L thiobarbituric acid were added, mixed well, and boiled for 15 min. The absorbance was measured at 535 nm. The hydroxyl radical scavenging activity was computed using Equation (2):

(2) OH• scavenging activity %=Acontrol−AsampleAcontrol−Aempty×100%

where $A_{sample}$ refers to the absorbance measured with the samples, $A_{control}$ represents the absorbance with deionized water in place of the samples, and $A_{empty}$ is the absorbance without the samples. ^•^OH (Inulin) represents the hydroxyl radical scavenging activity of the isolated inulin fraction, while ^•^OH (TPC) indicates the scavenging capacity associated specifically with the polyphenol fraction.

### 2.6. Assessment of Antioxidative Enzyme Activities

Frozen samples (5 g) were powdered under liquid nitrogen using pre-cooled mortar and pestle, maintaining low temperature throughout the grinding process, and enzymes were extracted. POD was extracted using 100 mM potassium phosphate buffer (pH 7.6) containing 1% (*v/v*) Triton X-100 and 4% (*w/v*) polyvinylpolypyrrolidone (PVPP), while CAT and SOD were extracted using 100 mM phosphate buffer (pH 7.6) containing 5% (*w/v*) polyvinylpyrrolidone (PVP) and 5 mM dithiothreitol (DTT) at 4 °C. Supernatants were collected after centrifugation at 12,000× *g* for 30 min at 4 °C.

POD and CAT activities were measured using a modified Maehly and Chance (1954) method [25]. For POD, 3 mL of 25 mM guaiacol, 0.1 mL enzyme extract, and 0.2 mL of 0.5 M H_2_O_2_ were used, with one unit (U) defined as a 0.1 increase in absorbance at 470 nm per minute. CAT activity was measured using 2.9 mL of 20 mM H_2_O_2_, with one unit (U) defined as a 0.01 decrease in absorbance at 240 nm per minute.

SOD activity, assessed following Pasquariello et al. (2015) [26], used a reaction mixture of 50 mM phosphate buffer (pH 7.6), 13 mM methionine (MET), 0.75 mM nitro-blue-tetrazolium (NBT), 0.1 mM EDTA-Na_2_, enzyme extract, and 0.02 mM riboflavin. The mixture was illuminated by a 40 W fluorescent lamp (emission spectrum 400–700 nm) for 15 min, and absorbance was recorded at 560 nm. One unit (U) was defined as the amount of enzyme required to achieve 50% inhibition of NBT photoreduction.

### 2.7. Statistical Data Analysis

The experiment employed a completely randomized design, primarily involving field trial area layout, selection of tubers for analysis, and the order of sample processing and analysis. Randomization was achieved through random number generation for plot assignment and systematic random sampling for tuber selection. All experimental data are expressed on a fresh weight basis except inulin, which is reported on a dry weight basis, and are presented as mean values accompanied by their respective standard errors (mean ± SE). To determine statistical differences among the treatments, the data undergo a one-way analysis of variance (ANOVA). This is followed by Duncan’s multiple range test, which is employed to identify significant differences between means, with statistical significance established at the *p* < 0.05 level. Significant differences are indicated by different letters for clarity. All statistical computations are conducted using SPSS Statistics Version 20.0 software. Additionally, visual representations of the data are created using Origin 9.0 software to enhance the interpretation of the results.

## 3. Results and Discussion

### 3.1. Inulin and Antioxidant Capacity

**Figure 1:**
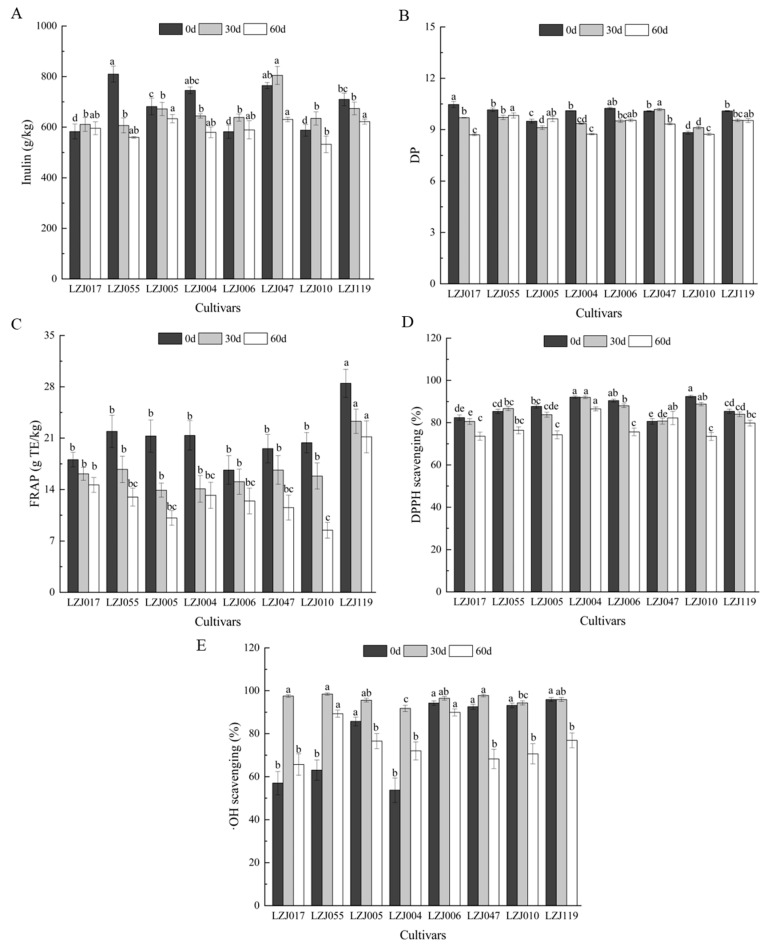
Changes in inulin content (**A**), DP (**B**), FRAP (**C**), DPPH scavenging ability (**D**) and •OH scavenging activity (**E**) in different varieties Jerusalem artichoke tubers during storage. Data are presented as mean ± SE (*n* = 3). Data with different letters are significantly different (*p* < 0.05).

Inulin content and its polymerization profile in Jerusalem artichoke tubers are critical indicators of their potential for both food and industrial applications. In particular, understanding the variations in inulin content, degree of polymerization (DP), and antioxidant capacity is essential for optimizing the functional and nutritional uses of these tubers. In this study, these parameters are monitored across eight cultivars during storage, revealing their dynamic changes over time (Figure 1, Supplementary Table S1). Fresh tubers exhibited inulin content ranging from 582.43 g/kg (LZJ006) to 809.70 g/kg (LZJ055). During storage, inulin content generally decreased in cultivars LZJ055, LZJ005, LZJ004, and LZJ119, while cultivars LZJ017, LZJ006, LZJ047, and LZJ010 saw an initial increase in inulin content during the first 30 days, followed by a decline. Notably, LZJ047 consistently maintained the highest inulin content, while LZJ010 showed the lowest across the storage period (Figure 1A). The DP of inulin also varied among cultivars, with LZJ017, LZJ004, LZJ006, and LZJ119 showing a continuous decrease throughout storage. Conversely, LZJ055 and LZJ005 initially decreased in DP up to 30 days before showing relative stability or slight increases between days 30–60 of storage (Figure 1B).

The decline in inulin content and DP is closely tied to the tubers’ metabolic activity during storage. This reduction is likely driven by respiratory consumption of reducing sugars and the hydrolytic breakdown of inulin by fructan: fructan 1-fructosyltransferase (1-FFT), an enzyme responsible for inulin depolymerization [27]. The breakdown of inulin into shorter-chain polymers releases free fructose, which serves several physiological roles, such as cryoprotection, by lowering the freezing point of cellular water and stabilizing cell membranes [28,29,30]. Additionally, fructose may act as an osmotic buffer, preventing cellular leakage and maintaining integrity under cold stress conditions [31].

A key innovative aspect of these findings is its focus on the pivotal role of inulin’s DP in determining its antioxidant capacity. While the overall structure of inulin is well-established, our results demonstrate that higher degrees of polymerization correspond to enhanced antioxidant activity. This finding highlights the importance of focusing on DP as a key determinant of inulin’s bioactivity, particularly in storage conditions where maintaining functional efficacy is critical.

In parallel to changes in inulin, antioxidant capacity showed cultivar-dependent variation throughout storage. For example, FRAP values uniformly decreased across all cultivars (Figure 1C), with LZJ119 consistently maintaining the highest antioxidant potential, while LZJ110 demonstrated the lowest FRAP value by the end of storage (8.5 ± 1.1 g TE kg^−1^). DPPH values similarly declined over time for most cultivars, except for LZJ055 and LZJ047, which displayed relatively stable DPPH antioxidant capacity (Figure 1D). In terms of •OH scavenging activity, all cultivars showed an increase during the first 30 days, followed by a gradual decline, with LZJ006 exhibiting the highest activity throughout storage, while LZJ004 displayed the lowest (Figure 1E).

Previous research has demonstrated that Jerusalem artichoke tubers, with their high inulin content, are a rich yet underutilized source of antioxidants and prebiotics for the functional food market [32,33,34]. The current study extends these findings by elucidating the complex relationship between inulin content, DP, and antioxidant capacity under cold storage conditions. The breakdown of inulin leads to increased availability of free fructose, which not only serves a protective role for the plant cells but also reinforces the plant’s antioxidant defense mechanisms against oxidative stress caused by low temperatures [35,36]. This suggests that inulin and its breakdown products play a multifaceted role in modulating the tubers’ resistance to oxidative stress during storage, with certain cultivars showing enhanced resilience due to their higher inulin content and antioxidant capacity.

Understanding these biochemical processes—especially the link between inulin content, DP, and antioxidant activity—is essential for improving the storage quality of Jerusalem artichoke tubers. By focusing on the functional role of inulin’s DP, this study provides a new perspective on the factors influencing the bioactive properties of Jerusalem artichoke tubers, paving the way for targeted breeding programs and postharvest management strategies.

### 3.2. Total Polyphenols Content and Antioxidant Capacity

**Figure 2:**
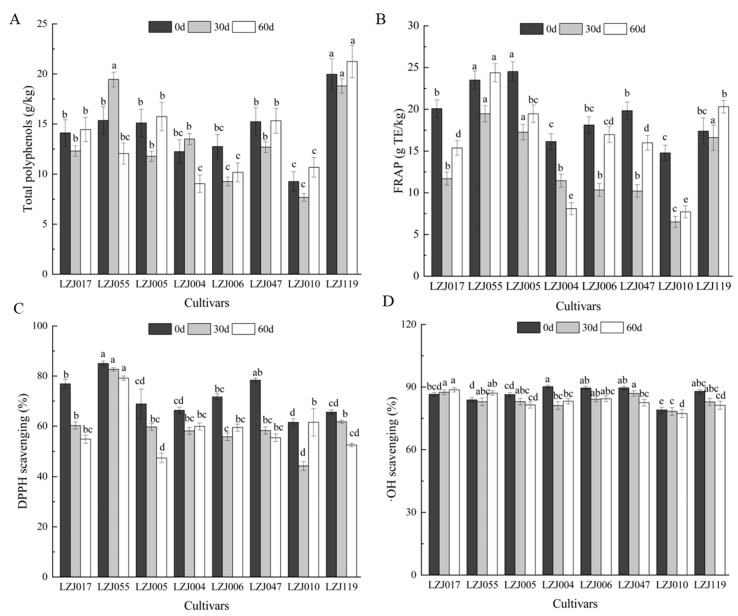
Changes in total polyphenol (**A**), FRAP (**B**), DPPH scavenging ability (**C**) and •OH scavenging activity (**D**) in different varieties Jerusalem artichoke tubers during storage. Data are presented as mean ± SE (*n* = 3). Data with different letters are significantly different (*p* < 0.05).

Polyphenols, as significant bioactive compounds in fruits and vegetables, play a key role in shaping their appearance, flavor, maturation, and senescence [37,38]. They also possess potent antimicrobial properties and are vital for scavenging free radicals, contributing significantly to the antioxidant capacity of plant-based foods. The polyphenol content of Jerusalem artichoke tubers varies depending on the cultivar, growth conditions, and storage environments [39]. In this study, most cultivars exhibited a gradual decrease in total polyphenol content during the first 30 days of storage, followed by an increase thereafter, with LZJ055 and LZJ004 showing distinct trends (Figure 2A, Supplementary Table S2). It should be noted that the Folin–Ciocalteu method can detect various non-phenolic reducing agents, and the values reported here may include contributions from other reducing compounds present in the tubers. Among all cultivars, LZJ119 consistently demonstrated the highest polyphenol content, whereas LZJ010 exhibited the lowest. Similar trends were observed in FRAP values for most cultivars, except for LZJ004 (Figure 2B). DPPH levels markedly decreased across all cultivars during the first 30 days of storage (Figure 2C), after which LZJ004, LZJ006, and LZJ010 showed an increase, while other cultivars continued to decline. LZJ055 had significantly higher DPPH levels (*p* < 0.05) compared to other cultivars, whereas LZJ010 displayed the lowest DPPH values. Regarding •OH scavenging activity, most cultivars exhibited a gradual decrease, except for LZJ017, LZJ055, and LZJ004, which maintained relatively stable levels throughout storage (Figure 2D).

The results revealed substantial differences in polyphenol content and antioxidant capacity across the different cultivars. Notably, LZJ119 contained 2.17 times more polyphenols than LZJ010. The majority of cultivars exhibited a decrease in polyphenol content prior to 30 days, followed by a subsequent increase, suggesting that polyphenol accumulation may be influenced by the plant’s response to cold stress or other postharvest storage conditions [40]. Such trends have been observed in other crops as well, such as apples, tomatoes, and potatoes, where polyphenol accumulation has been linked to abiotic stress factors, allowing for the differentiation between resistant and susceptible cultivars [41,42].

Polyphenols, through their well-documented antioxidant properties, actively participate in neutralizing reactive oxygen species (ROS) such as O2^• −^, H_2_O_2_, and hydroxyl radicals, which are commonly generated during cold storage. Although low-temperature storage effectively slows down metabolic activity and reduces decay, it simultaneously promotes the production of ROS, which can deplete antioxidants through redox reactions. The antioxidant role of polyphenols is primarily mediated through hydrogen atom donation, peroxide breakdown, and the prevention of lipid peroxidation by scavenging lipid alkoxyl radicals [43].

The decline in polyphenol content and antioxidant capacity observed in most Jerusalem artichoke cultivars may be closely linked to their active involvement in ROS scavenging. As ROS are neutralized by polyphenols, these bioactive compounds are consumed, leading to a reduction in both polyphenol content and overall antioxidant capacity. This relationship underscores the role of polyphenols in the plant’s oxidative stress response during storage, where their depletion reflects their continuous interaction with ROS. Consequently, the observed reduction in antioxidant capacity in most cultivars is likely a direct result of the active participation of polyphenols in mitigating oxidative stress.

A novel aspect of this study is its examination of the interaction between inulin and polyphenols in the antioxidant system of Jerusalem artichoke tubers. Inulin degradation during storage releases free fructose, which may act synergistically with polyphenols to enhance the antioxidant defenses of the tubers. This interplay between inulin and polyphenols could be a critical factor in determining the tubers’ resistance to oxidative damage and their overall antioxidant potential throughout the storage period. Understanding this dynamic is essential for optimizing the storage and utilization of Jerusalem artichoke tubers in various food and industrial applications.

### 3.3. Antioxidant Enzymes Activities

**Figure 3:**
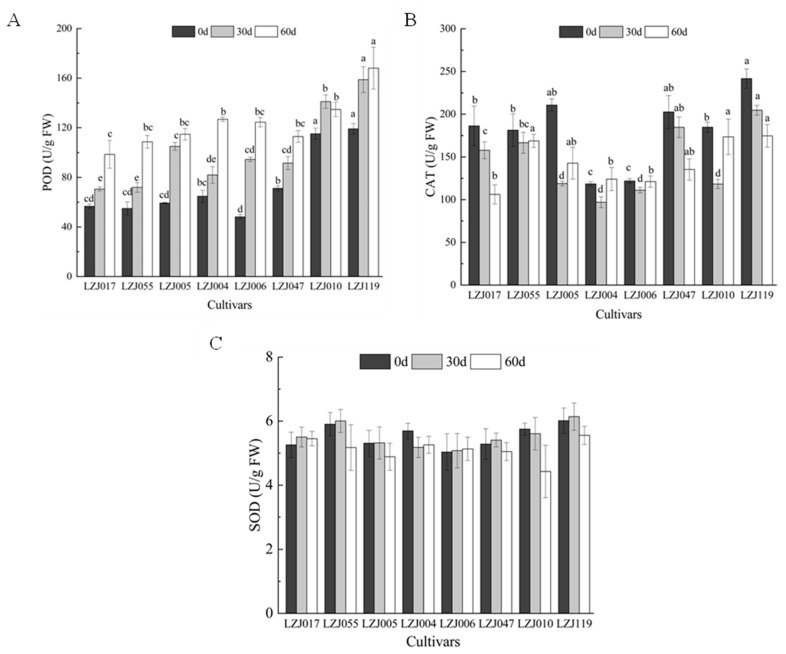
Changes of POD (**A**), CAT (**B**) and SOD (**C**) activities in different varieties of Jerusalem artichoke tubers during storage. Data are presented as mean ± SE (*n* = 3). Data with different letters are significantly different (*p* < 0.05).

POD activity showed significant variations across all eight cultivars during cold storage (Figure 3A, Supplementary Table S3). Statistical analysis revealed significant differences in the magnitude of POD activity changes among cultivars (*p* < 0.05). LZJ119 exhibited the highest POD activity, followed closely by LZJ010, while LZJ017 recorded the lowest. In contrast, CAT activity showed a more variable pattern (Figure 3B). In cultivars LZJ017, LZJ047, and LZJ119, CAT activity steadily decreased throughout the storage period. However, in LZJ055, LZJ005, LZJ004, LZJ006, and LZJ010, CAT activity showed a V-shaped pattern, with a significant decline during the first 30 days of storage (*p* < 0.05), followed by an increase towards the end of the storage period. Among all cultivars, CAT activity was significantly higher in LZJ047 and LZJ119, while it was lower in LZJ004 and LZJ006. As for SOD activity (Figure 3C), there were no significant differences among the cultivars at most time points (*p* > 0.05). Generally, SOD activity increased initially and then declined, with the exception of LZJ004, LZJ006, and LZJ010, which showed different patterns, though these differences were not always statistically significant.

The activity of antioxidant enzymes such as SOD, POD, and CAT plays a vital role in neutralizing free radicals and mitigating oxidative stress. These enzymes act in concert to regulate reactive oxygen species (ROS) levels, preventing excessive oxidative damage to cellular structures during cold storage. Under low-temperature conditions, the accumulation of ROS is influenced by various factors, including oxygen levels, physical damage, microbial infections, and natural aging processes. Elevated ROS levels can lead to membrane lipid peroxidation, which compromises cellular integrity and decreases the storability of tubers [44,45]. In this study, the consistent increase in SOD and POD activities across most cultivars suggests an adaptive response to the rising ROS levels during storage. In contrast, the initial rise and subsequent decline in CAT activity could indicate enzyme depletion or regulation mechanisms as storage progresses. The fluctuations in antioxidant enzyme activities are critical for maintaining ROS balance, thereby protecting the tubers from extensive oxidative damage.

This study sheds new light on the role of inulin in the antioxidant system of Jerusalem artichoke tubers. As a key carbohydrate reserve, inulin breakdown during storage not only provides energy but also releases free fructose, which may further support antioxidant activity [30,35]. This process could enhance the performance of antioxidant enzymes by modulating osmotic balance and preventing oxidative stress. For instance, cultivars with higher inulin content, such as LZJ119 and LZJ047, also showed higher CAT and POD activities, suggesting a potential interaction between inulin degradation products and the regulation of antioxidant enzyme activities. This novel finding highlights the importance of inulin in bolstering the tubers’ resilience against oxidative stress during extended cold storage.

Among all the cultivars studied, LZJ119 stood out with the highest antioxidant enzyme activities, indicating a superior capacity to counteract ROS accumulation. This enhanced antioxidant response likely contributes to its improved storage quality and could be a result of its genetic stability, potentially enhanced through tissue culture propagation. LZJ119’s high yield and adaptability to the semi-arid conditions of the Loess Plateau further support its suitability for prolonged cold storage.

### 3.4. Hierarchical Cluster Analysis of Antioxidant Parameters and Enzymatic Activities

**Figure 4:**
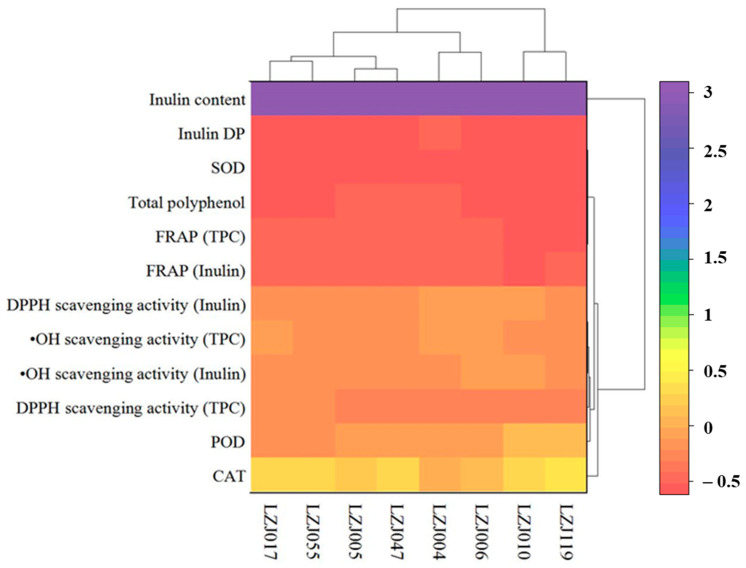
Hierarchical cluster analysis of antioxidant enzyme activities, antioxidant capacity measurements, and related parameters in different varieties of Jerusalem artichoke tubers.

A two-way hierarchical cluster analysis (HCA), complemented by a heatmap, is presented to assess the comprehensive antioxidant profiles of the different Jerusalem artichoke cultivars, including antioxidant enzyme activities (SOD, CAT, POD), antioxidant capacity measurements (FRAP, DPPH, ^•^OH scavenging), and related parameters (inulin content, DP, and polyphenol content). This analysis reveals three distinct clusters among the eight accessions, reflecting the variability in their antioxidant capacities (Figure 4). Based on the integrated analysis of antioxidant enzyme activities and related antioxidant parameters, the cultivars were grouped into three unique clusters. Cluster I, which exhibited the highest antioxidant capacity, included cultivars LZJ119 and LZJ010. These cultivars consistently showed superior antioxidant performance across all measured parameters. Cluster II, composed of LZJ006 and LZJ004, demonstrated moderate antioxidant capacity, while Cluster III, consisting of LZJ005, LZJ055, and LZJ017, was characterized by the lowest antioxidant activity among the cultivars.

The classification of these clusters provides important insights into the distinct antioxidant systems operating within the different cultivars of Jerusalem artichoke, particularly under the cold storage conditions of a semi-arid region in China. This analysis is critical for understanding the mechanisms driving variation in antioxidant capacity, which could guide breeding programs and postharvest management strategies to enhance the nutritional quality and storage potential of these tubers.

Notably, the heatmap generated from the HCA underscored that inulin content and its degree of polymerization (DP) were the most significant factors contributing to the differentiation among the clusters. The pivotal role of inulin in distinguishing the antioxidant profiles of the cultivars highlights its influence on the tubers’ ability to mitigate oxidative stress during storage. This novel finding suggests that cultivars with higher inulin content and optimized DP, such as LZJ119, are more adept at maintaining antioxidant capacity, likely due to the functional benefits provided by inulin and its breakdown products, such as free fructose. These inulin-related traits were key drivers of clustering, while other factors appeared to have a lesser impact on the overall grouping. The differential clustering of cultivars based on inulin content and DP further suggests that selecting cultivars with optimal inulin characteristics could enhance their postharvest resilience, particularly in regions subjected to cold stress. Consequently, inulin and its related traits should be prioritized in future breeding efforts aimed at improving the storage quality and antioxidant capacity of Jerusalem artichoke.

### 3.5. Relationship Between the Antioxidant and the Antioxidant Activity

**Figure 5:**
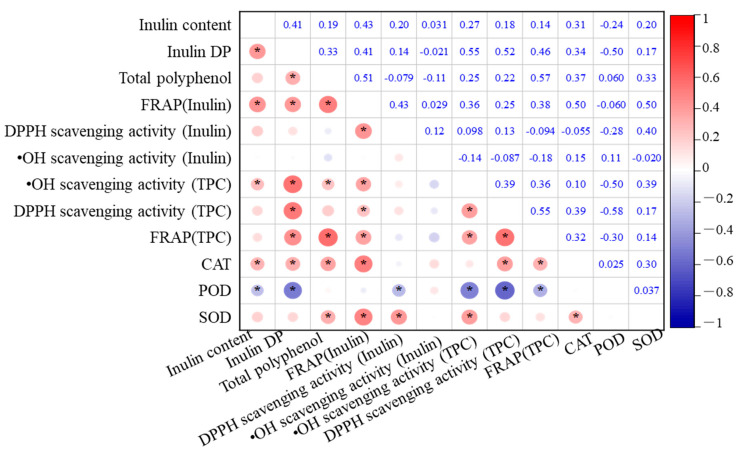
Correlation matrix of antioxidant capacity and antioxidant systems in Jerusalem artichoke cultivars during cold storage in a semi-arid region of China. * indicates statistical significance at *p* < 0.05.

The relationships among total polyphenol content (TPC), inulin content, antioxidant enzyme activities, and antioxidant capacity in the eight Jerusalem artichoke cultivars are analyzed, as depicted in Figure 5. Inulin content exhibited significant positive correlations with the FRAP of inulin, DP, and CAT activity (*p* < 0.01). This highlights the central role of inulin in enhancing the antioxidant capacity of the tubers. Higher inulin content not only contributes to the FRAP values but also supports enzymatic defense systems, particularly CAT, which is crucial for decomposing hydrogen peroxide into water and oxygen, thus preventing oxidative damage.

In addition, DP was positively correlated with TPC, FRAP of inulin, •OH scavenging activity, DPPH scavenging activity, and CAT (*p* < 0.01), indicating that the degree of inulin polymerization significantly enhances the antioxidant potential of the tubers. These correlations suggest that more polymerized inulin can stabilize cell structures and maintain osmotic balance under oxidative stress conditions, indirectly supporting polyphenols and antioxidant enzymes in scavenging free radicals.

TPC also exhibited strong positive correlations with the FRAP of inulin, overall FRAP, and CAT activity (*p* < 0.01). This implies that polyphenols and inulin synergistically contribute to the antioxidant system, with polyphenols playing a direct role in scavenging ROS, while inulin supports the enzymatic antioxidant defense by maintaining cellular homeostasis. The combined effect of polyphenols and inulin enhances the overall reducing power of the tubers, further protecting them from oxidative damage during storage.

Significant positive correlations were also observed between CAT, FRAP of inulin, DPPH scavenging activity, and overall FRAP. Additionally, SOD displayed significant correlations with FRAP of inulin, DPPH scavenging activity of inulin, and •OH scavenging activity (*p* < 0.01), suggesting that SOD plays a complementary role to inulin in mitigating oxidative stress, particularly by converting superoxide radicals into less reactive molecules.

Conversely, POD activity showed a negative correlation with DP, •OH scavenging activity, and DPPH scavenging activity (*p* < 0.01). This negative correlation suggests that in cultivars with higher inulin polymerization, POD activity may not be as critical in antioxidant defense, possibly because inulin and CAT provide more efficient protection against oxidative stress. This shift in the role of antioxidant enzymes depending on inulin levels highlights the importance of understanding how different cultivars optimize their antioxidant systems.

Although inulin and other sugars such as fructans and sucrose contribute to maintaining osmotic balance and cellular membrane integrity, particularly under stress conditions like salinity, their exact role in balancing the redox status in cells and neutralizing ROS is not fully clear. Research by Stoyanova et al. (2011) suggests that these sugars help preserve osmotic balance and protect cell membranes during salinity stress, which could also apply under cold storage conditions [46]. The ability of mild stress to amplify tuber fructan content further supports the idea that inulin plays a critical role in helping plants manage environmental stressors.

The relationship between inulin and antioxidant activity in Jerusalem artichoke tubers could provide valuable insights into optimizing inulin extraction from tubers cultivated in different regions, such as Northeastern China, where environmental stresses may affect inulin content and structure. The enhanced inulin content under certain stress conditions, such as salinity or low temperature, suggests that inulin could be selectively extracted to improve its antioxidant properties for use in functional foods and other applications.

Interestingly, this mechanism mirrors what has been observed in other antioxidant-rich plants, such as pomegranates, which demonstrate strong antioxidant capacity primarily due to high polyphenol concentrations, including flavonoids and anthocyanins [47,48]. As inulin, polyphenols, and antioxidant enzymes accumulate in the vacuoles of Jerusalem artichoke tubers, where salts also tend to accumulate, it is hypothesized that this compartmentalization serves as a defense mechanism against stress-induced oxidative damage [49]. Under stress, ROS could act as signaling molecules, triggering the production of more antioxidants, including inulin, which would help maintain redox balance and protect the plant from further damage. By understanding the interplay between inulin, polyphenols, and antioxidant enzymes, future efforts could focus on enhancing the tubers’ functional properties through selective breeding and optimized postharvest storage strategies.

## 4. Conclusions

This study demonstrates the critical role of inulin in regulating the antioxidant capacity and maintaining the antioxidant system of Jerusalem artichoke tubers during cold storage. A reduction in inulin content and its degree of polymerization (DP) was observed over 60 days, indicating inulin’s susceptibility to cold conditions. The significant correlations between inulin, polyphenols, and antioxidant enzymes such as CAT, SOD, and POD confirm that inulin not only serves as a carbohydrate reserve but also plays a pivotal role in sustaining the tubers’ antioxidant system. By stabilizing cellular structures and enhancing enzymatic defenses, inulin substantially contributes to the overall antioxidant capacity. Future research should focus on optimizing inulin stability to further enhance the functional and nutritional benefits of Jerusalem artichokes.

## Data Availability

The original contributions presented in this study are included in the article. Further inquiries can be directed to the corresponding author(s).

## Supplementary Materials

The following supporting information can be downloaded at: https://www.mdpi.com/article/10.3390/antiox14091109/s1, Supplementary Table S1: Changes in inulin content, degree of polymerization (DP), and associated antioxidant capacities (DPPH scavenging, •OH scavenging, and FRAP); Supplementary Table S2: Changes in total polyphenol content and polyphenol-associated antioxidant capacities (DPPH scavenging, •OH scavenging, and FRAP); Supplementary Table S3: Changes in antioxidant enzyme activities (CAT, POD, and SOD).
